# Methane emissions from macrophyte beach wrack on Baltic seashores

**DOI:** 10.1007/s13280-022-01774-4

**Published:** 2022-08-27

**Authors:** Mats Björk, Gunilla Rosenqvist, Fredrik Gröndahl, Stefano Bonaglia

**Affiliations:** 1 Department of Ecology, Environment and Plant Sciences, Stockholm University, 106 91 Stockholm, Sweden; 2 Blue Centre Gotland, Uppsala University-Campus Gotland, 621 67 Visby, Sweden; 3 KTH, Royale Institute of Technology, KTH Teknikringen 10B, Stockholm, Sweden; 4 Department of Sustainable Development, Environmental Science and Engineering, 100 44 Stockholm, Sweden; 5 Department of Marine Sciences, University of Gothenburg, Box 461, 405 30 Gothenburg, Sweden

**Keywords:** Beach cast, Baltic Sea, Climate change, Greenhouse gas, Marine macrophytes, Methane

## Abstract

Beach wrack of marine macrophytes is a natural component of many beaches. To test if such wrack emits the potent greenhouse gas methane, field measurements were made at different seasons on beach wrack depositions of different ages, exposure, and distance from the water. Methane emissions varied greatly, from 0 to 176 mg CH_4_-C m^−2^ day^−1^, with a clear positive correlation between emission and temperature. Dry wrack had lower emissions than wet. Using temperature data from 2016 to 2020, seasonal changes in fluxes were calculated for a natural wrack accumulation area. Such calculated average emissions were close to zero during winter, but peaked in summer, with very high emissions when daily temperatures exceeded 20 °C. We conclude that waterlogged beach wrack significantly contributes to greenhouse gas emissions and that emissions might drastically increase with increasing global temperatures. When beach wrack is collected into heaps away from the water, the emissions are however close to zero.

**Supplementary Information:**

The online version contains supplementary material available at 10.1007/s13280-022-01774-4.

## Introduction

Beach wrack is any natural material that washes and accumulates onto a shore, typically consisting of a variety of algae, seagrasses, and invertebrates. In undisturbed systems, the wrack fills many ecological functions. For example, it provides nutrients, represents an important habitat and food source for many organisms, and also acts as a natural barrier to large waves (Kirkman and Kendrick 1998). Traditionally, wracks have been highly valued by farmers, who have harvested and used them as organic fertilizers (Villares et al. 2016). It is in the nature of beach wrack to constantly change in mass and composition and to be brought to the shore depending on winds and currents. On Baltic seashores, the increasing level of eutrophication has altered the composition of the beach wrack, with an increased proportion of thinner and filamentous macroalgae, making the beach wrack denser and more nutrient rich (Chubarenko et al. 2021). At the German Baltic Sea coast, the accumulated beach wrack biomass has increased by a factor of 3.4 between 1977 and 2013, while the composition changed toward a larger proportion of ephemeral and nutrient-opportunistic seaweeds (Weinberger et al. 2021). This has posed a problem as beaches have become increasingly fouled and the larger accumulations of beach wrack has resulted in significantly reduced recreational values of such coastal areas (Stenis et al. 2021). As a result, there has been a considerable “Willingness To Pay” (WTP) among local residents along the Baltic Sea to regularly remove beach wrack algae (Risen et al. 2017). In some Baltic coastal regions, e.g., the Swedish communities in Gotland, Trelleborg, and Laholm, beach wrack is regularly removed from beaches during the tourist season. The wrack is gathered in heaps and often subsequently used on agricultural lands. In Sweden, such activities can be partly funded from the Swedish Agency for Marine and Water Management who funds activities that reduce eutrophication in the Baltic Sea (SFS 2009, p. 381); the funds on Gotland is distributed by the County Administration Board of Gotland.

Methane (CH_4_) is a potent greenhouse gas with a global warming potential (on a 100-year time scale) exceeding that of carbon dioxide (CO_2_) by factors of 28–34 (Myhre et al. 2013). It is estimated that nearly 40% of the total methane emission to the atmosphere comes from natural systems (Scheehle and Kruger 2003; Saunois et al. 2020). Presently, the contribution of submerged macrophytes to global estimates of such greenhouse gases is increasingly investigated (Bahlmann et al. 2015; Garcias-Bonet and Duarte 2017) with an improved understanding of emissions from shallow coastal vegetated areas (Lyimo et al. 2018; Burkholz et al. 2020; George et al. 2020; Asplund et al. 2022; Roth et al. 2022). Beach wrack has been shown to have a high methane production potential in laboratory studies (Risen et al. 2014; Marquez et al. 2013; Mission et al. 2020), but few estimations of their net emissions in natural settings have been reported. Seagrass wrack from Mediterranean beaches emitted low levels of methane (Mission et al. 2020, 2021), while seagrass wrack from beach deposits in Australia were found to emit no detectable methane but high levels of CO_2_ (Liu et al 2019). However, in an overview of options for dealing with excessive beach wrack, Pal and Hogland (2022) suggest that compact beach wrack left on beaches might be a significant emitter of methane and other greenhouse gases. Given the large potential of beach wrack to produce methane and that methane is positively correlated to both temperature and humidity (Borges et al. 2018) it is important to study how emissions might change under different environmental conditions.

Marine macrophyte systems fix CO_2_ in photosynthesis and store large amounts of carbon. They are therefore considered effective sinks for atmospheric CO_2_ with a good climate mitigation potential (Mcleod et al. 2011), but if the biomass production from these systems ends up as a compact and dense beach wrack as described for eutrophic systems (Tatarchenko 2011), their carbon sink function may be partly counteracted by associated increases in methane emissions. The aims of the present study were therefore to: (i) estimate methane emissions from macrophyte beach wrack of different water contents, age, and biomass density during multiple seasons, (ii) at the same time evaluate possible effects on these emissions after their removal from the beach waterfront to compost heaps, and (iii) extrapolate the measured methane emissions to build an annual-scale budget. We investigated emissions with a combination of field and laboratory experiments and used the eutrophic Baltic Sea as a model system.

## Materials and methods

### Study area

**Fig. 1:**
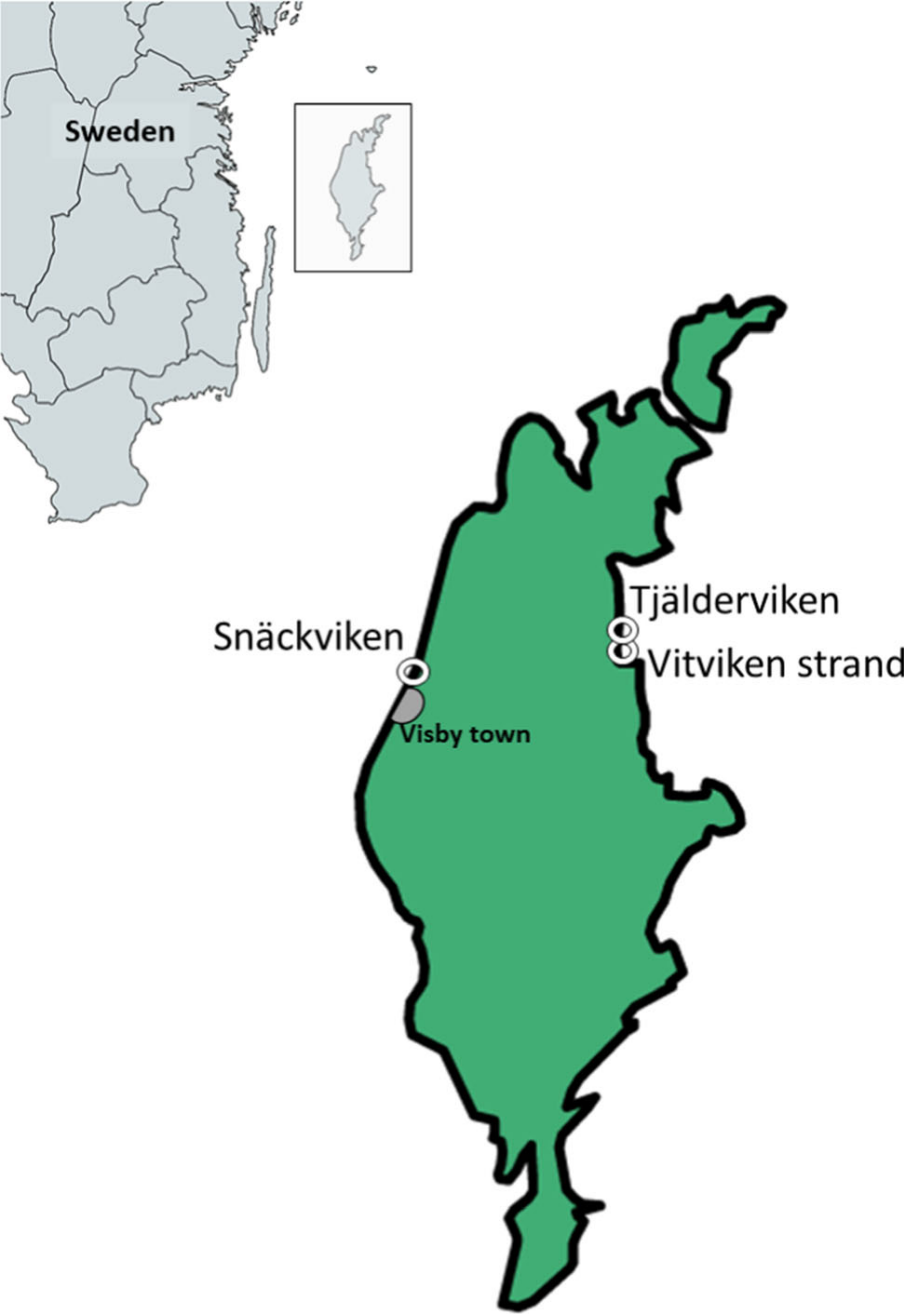
Map of the island of Gotland with the main sampling sites indicated by circles. (Map created using mapchart.net)

This study was conducted at the Island of Gotland situated in the middle of the Baltic Sea (Sweden). We selected three different bays on the Island where beach wrack is frequently found: Tjälderviken and Vitviken on the east coast and Snäckviken on the west coast (Fig. 1).

**Fig. 2:**
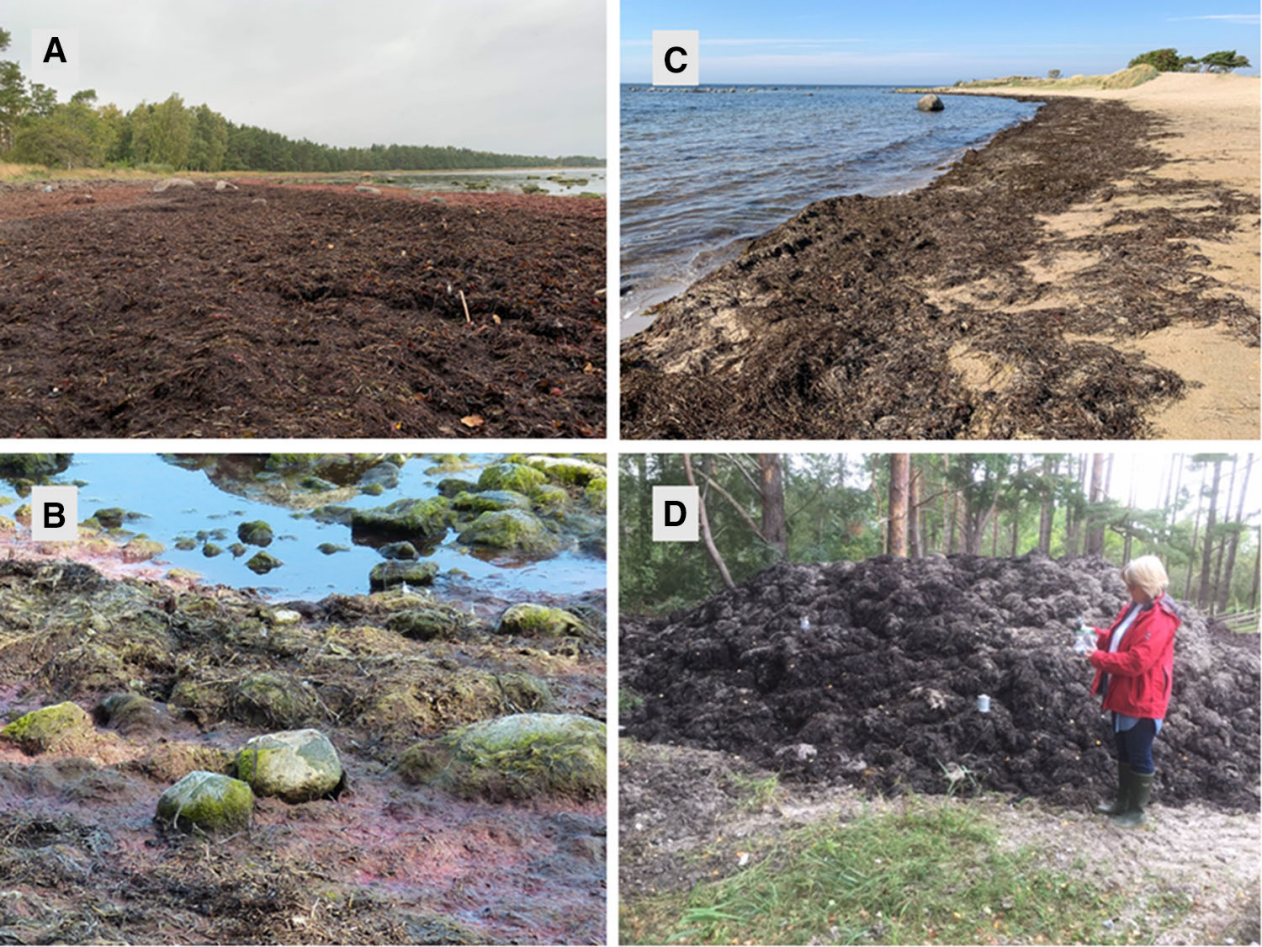
Accumulating beach wrack at the sheltered beach site Tjälderviken (**a** and **b**), fresh wrack at the exposed sandy beach Snäckviken (**c**), and wrack collection heap at site Vitviken (**d**). (Pictures by the authors)

Tjälderviken (N 57.632476, E 18.765809) was selected as the main site for repeated field measurements as it is an easily accessible, remote, and sheltered pebble/stone beach where the wrack is not collected by the municipality (Fig. 2a, b). Thus the beach wrack accumulates over long time periods and is continuously found in different stages of decomposition.

Snäckviken (N 57.674267, E 18.331839) is a coarse sandy beach exposed to waves and wind. The bay is used in the summer for recreation and is cleaned every year from beach wrack. Even though wrack is regularly deposited on the beach by waves and wind, it seldom accumulates for any longer time at this site (Fig. 2c).

Vitviken strand (N 57.622933, E 18.765809) is a sandy beach exposed to wind mostly from north–east and east. The beach area is cleaned regularly from beach wrack as the bay is used as a popular sandy beach by tourists; thus beach wrack is seldom accumulating for any longer time at this site.

For comparisons, three wrack collection sites were also chosen. At those sites, beach wrack was collected from the nearest beach and put into heaps, approx. 3–6 m in diameter and 1.5–3 m high (Fig. 2d) to later be transported to agricultural sites to be used as fertilizer. The three wrack collection sites were Vitviken, Midsommarvägen, and Röcklinge. Beach wrack are collected from adjacent beaches and stored in heaps at Vitviken and Midsommarvägen, with biomass staying 1–2 months in the heap before being distributed over farmland. In Röcklinge beach wrack was collected on standard garden mesh containers. A typical collection heap (Vitviken) is depicted in Fig. 2d.

### Methane (CH_4_) sampling in the field

Gas fluxes across the wrack–air interface were measured by sampling air in the headspace of plastic chambers placed into the wrack over periods of approximately 24 h. The static open chamber method was chosen as most suitable for the wrack environment. The sites for sampling of gas emissions were chosen to represent the wrack area close to the water (moister) and the area away from the water (drier). These areas show an increasing dryness of the wrack, from the water up toward land. Sampling was performed in the following order: (1) At day 1, between 10:00 and 12:00, plastic chambers (∅ 13 cm, height 12 cm; chamber volume, 0.80 L) equipped with a rubber septum were placed 2–3 cm deep into the wrack and two gas subsamples were immediately collected from each chamber using a gas-tight syringe and transferred to a pre-evacuated 5.9 mL Exetainer (Labco Limited). (2) At day 1, between 14:00 and 16:00, two gas subsamples were collected from each chamber. (3) At day 2, between 10:00 and 12:00, two gas subsamples were collected from each chamber and the chambers were collected. The gas-filled Exetainers were kept cold and dark while transported to the laboratory for analysis, which was performed within two weeks.

Sampling dates are given in Table 1. Samples from the ambient air about 2 m from the wrack were taken before commencing the measurements at all sites to eliminate possible errors due to other methane sources.

**Table 1:** Methane emissions from beach wrack estimated at the different sites. Values are averages (± SD) for each sampling site

	Description	Type	Times sampled	Dates sampled	Temp (ºC)	Overall CH_4_ flux (mg CH_4_-C m^−2^ day^−1^)				
						Min	Max	average	*n*	*SE*
Main beach site										
Tjälderviken	Beach wrack, older decomposing layer	Beach, sheltered	5	June 24, 2020	18.8	*7.17*	*176.07*	*75.44*	*3*	*41.95*
				September 5, 2020	15.5	*0.00*	*34.64*	*3.90*	*9*	*3.39*
				September 15, 2020	16.6	*0.88*	*53.86*	*17.25*	*4*	*10.71*
				December 5, 2020	7.3	*0.00*	*13.79*	*2.31*	*6*	*2.10*
				March 6, 2021	1.7	*0.00*	*0.00*	*0.00*	*5*	*0.00*
				***All samples***		**0.00**	**176.07**	**12.75**	***27***	***6.71***
Auxiliary beach sites										
Vitviken strand	Beach wrack, newly deposited layer	Beach, exposed	1	September 15, 2020	22.0					
				***All samples***		**0.01**	**0.33**	**0.19**	***4***	***0.07***
Snäckviken	Beach wrack, newly deposited, living material	Beach, exposed	1	September 15, 2020	20.5					
				***All samples***		**0.00**	**0.01**	**0.00**	***6***	***0.00***
Collection sites										
Vitviken	Compost heap on the upper beach	Collection site, 50 m from beach	2	June 24, 2020	23.6	*0.00*	*0.00*	*0.00*	*2*	*0.00*
				September 5, 2020	16.7	*0.09*	*0.36*	*0.23*	*2*	*0.10*
				***All samples***		**0.00**	**0.36**	**0.11**	***4***	***0.07***
Midsommarvägen	Compost heap on the upper beach	Collection site	1	December 5, 2020	8.3					
				***All samples***		**0.00**	**0.01**	**0.00**	***3***	***0.00***
Röcklinge	Compost containers	Collection site	2	June 24, 2020	24.8	*0.00*	*0.07*	*0.03*	*4*	*0.01*
				September 15, 2020	20.5	*0.00*	*0.01*	*0.01*	*6*	*0.00*
				***All samples***		**0.00**	**0.07**	**0.02**	***10***	***0.01***

### Methane analysis

Gas samples from the Exetainers were analyzed using a gas chromatograph (GC 8A, Shimadzu Corporation) equipped with a Porapak N column (80–100 mesh) and a flame ionization detector (FID). The carrier gas and make-up gas were nitrogen, while the FID fuel gas was hydrogen plus air. For calibration, certified standards at atmospheric concentration (1.9 ppm) and with 49.9 ppm CH_4_ (AirLiquide Gas AB) were used.

### Methane emission computations

Using the Ideal Gas Law (PV = nRT), the ppm concentrations were converted into molar concentrations (µmol CH_4_ L^−1^), which were plotted against incubation time (day). The CH_4_ emissions from the macrophyte wrack were then calculated as the total amount of CH_4_ carbon (C) accumulating over time (day) within the chamber area (m^−2^) and reported as mg CH_4_-C m^−2^ day^−1^. Since the chambers did not have stirring mechanisms resembling wind, the measured emissions had to be considered as conservative estimates. The production of CH_4_ in lab experiments was calculated as the linear increase in CH_4_ over time (day) per gram of incubated beach wrack biomass (g) and was reported as mg CH_4_-C g^−1^ day^−1^.

### Estimations of wrack biomass, composition, and water content

A simple estimation on the biomass load was made by random measurements of the thickness and width of the wrack belts on the beach. The length of the covered beach was estimated using Google Earth (Imagery date: August 15, 2020). To estimate the composition and water content of the wrack, random samples were taken at different beach locations. The samples were sorted into six categories: brown algae/kelp, seagrass, red algae, green algae, animal remains, and unidentified material. When possible and depending on the degree of decomposition, samples were also identified down to genus or species level. These samples were then weighed and oven dried at 60 ºC until constant weight and re-weighed so as to calculate the water content.

### Temperature data

Average diel temperature data were downloaded from SMHI, the Swedish Meteorological and Hydrological Institute (https://www.smhi.se/data/), at the closest available station, which was station “Östergarnsholm A” (SMHI station code 78,280, N 57.4408, -E 18.9839) for the time Jan 2016 to December 2020.

### Calculation of estimated average emission of methane

The measured emissions at Tjälderviken (all samples) were plotted against the average diel temperature at the time of measurement. The equation of the exponential correlation (*y* = 2E−05*x*^5.0948^; *R*^2^ = 0.9529) between emissions and average diel temperature was then used to give an estimate of temperature-driven changes in methane emissions. We included here emission data from wrack of all water contents, from furthest from the water to the waterlogged wrack at the water level.

### Data on existing beach wrack accumulation and gathering on Gotland

Data were kindly supplied from County Administration Board of Gotland, and the data were collected from LOVA project rapports (funding to support removing nutrition from the Baltic Sea) from 2011 to 2015.

### Laboratory incubations and sampling

To examine if the beach wrack by itself had the capacity to produce methane, beach wrack close to the water was collected from Tjälderviken on the 16th of September 2020. Emission rates were then measured under controlled laboratory conditions. The material was incubated in 500 mL glass flasks and immersed into thermostat regulated water baths and then divided into three temperature treatments: 15, 20, or 25 °C, with six replicate samples in each. A duplicate gas sample was taken from each flask at start and after approx. 2, 3, 4, 20, and 50 h from start.

## Results

### Methane emission in the field

**Fig. 3:**
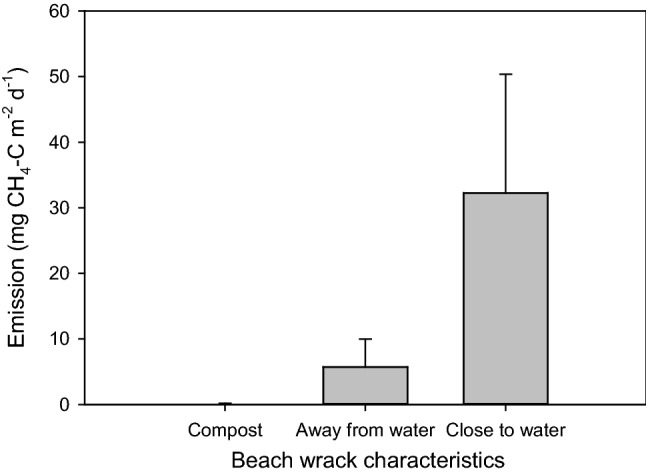
Methane (CH_4_) emissions in situ from the surface of beach wrack accumulated in Tjälderviken and the beach wrack compost/collection sites, showing average emissions grouped into the wet beach wrack closest to the water edge, the drier beach wrack higher up on the beach, and the emissions from the composts of beach wrack gathered in heaps in close connection to the beaches (data are averages + SE, *n* = 4–12). Measurements were performed in June and September. There was a statistically significant difference (Kruskal–Wallis; *P* = 0.046) in methane emissions between the three treatments

Overall the emissions of methane in the field investigations varied greatly (Table 1), ranging from zero mostly in the drier parts of the wrack to 176 mg CH_4_-C m^−2^ day^−1^ in the waterlogged part. As a general trend, the emissions appeared to be correlated with the water content (indicated in Table 2), so that wrack at the water line (with an estimated water content of about 90%) emitted the highest levels, while the drier wrack material higher up on the beach (estimated water content 50%) or in the compost heaps (estimated water content 15%) (Fig. 3) emitted lower levels, or no, methane. This was however not true for the very fresh (and wet) material measured at Snäckviken or Vitviken strand sites that emitted no, or very low levels, of methane (Table 1).

**Table 2:** Water content and the percentage of the different groups’ composition of wrack collected from beaches and compost heaps. The wrack was classified by visual identification. Values are averages (± SE) of the percentage of the total dry weight

		Water content (%)	Relative proportion of different taxa in the collected wrack (% of the total dry weight)						n/times sampled
			Brown algae	Red algae	Green algae	Seagrass)	Animals	Unidentified	
Main beach site:									
Tjälderviken, away from water	Beach wrack, older decomposing layer on average 55 ± 14 cm thick	50.8 ± 7.8	5.1 ± 4.1	8.3 ± 2.7	1.1 ± 0.4	26.8 ± 4.0	0.3 ± 0.1	58.3 ± 6.3	(6/2)
Tjälderviken, at waterfront	Beach wrack, older decomposing layer on average 31 ± 12 cm thick	87.7 ± 0.7	3.0 ± 1.5	53.8 ± 10.0	0.2 ± 0.1	13.3 ± 0.9	0.9 ± 0.2	28.9 ± 10.0	(30/2)
Auxiliary beach sites:									
Vitviken strand	Beach wrack, newly deposited layer on average 5 ± 3 cm thick, partly living material	48.9 ± 6.5	0.8 ± 0.4	15.8 ± 2.6	0.8 ± 0.2	7.8 ± 1.3	0.7 ± 0.2	74.1 ± 3.9	(6/1)
Snäckviken	Beach wrack, newly deposited layer on average 21 ± 5 cm thick, living material	63.6 ± 4.2	46.0 ± 9.8	34.9 ± 8.9	5.7 ± 1.5	3.6 ± 0.7	0.9 ± 0.7	9.0 ± 3.4	(5/1)
Slite strand	Beach wrack, older decomposing layer on average 13 ± 4 cm thick	75.8 ± 5.6	20.3 ± 14.2	1.6 ± 1.1	6.8 ± 2.7	17.3 ± 4.0	1.7 ± 0.4	52.3 ± 10.7	(5/1)
Collection sites:									
Slite badvik	Compost heaps (3 different) on the upper beach	15.0 ± 1.1	14.7 ± 9.4	7.1 ± 5.0	0.1 ± 0.1	5.7 ± 3.4	0.8 ± 0.2	71.6 ± 8.6	(5/1)

**Fig. 4:**
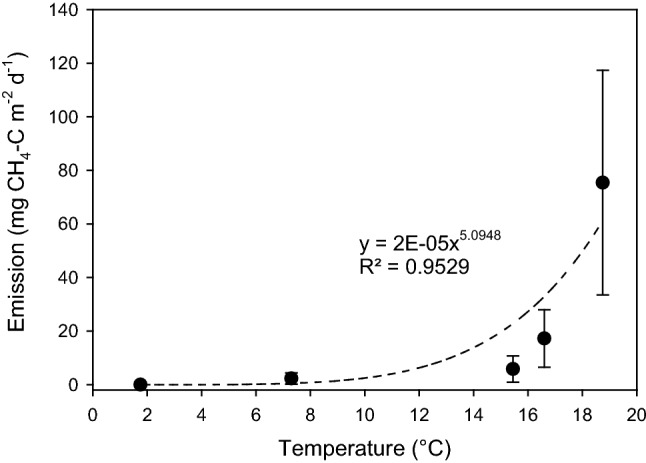
Temperature dependence of methane (CH_4_) emissions in situ from the surface of beach wrack accumulated at site Tjälderviken during different seasons. Values are averages (± SE, n = 3–9). Dates for gas emission measurements are given in Table 1

At Tjälderviken, where we sampled at different seasons and a range of temperatures, it was clear that emissions also were positively correlated with temperature (Fig. 4).

### Water content and composition of beach wrack

The water content of wrack varied from 15% (± 1.1SE) at the sampled compost heaps on the upper beach, to about 50% on the beach wrack on the upper parts of the beach and 88% (± 0.7SE) water in the wrack closer to the waterfront (Table 2). The wrack consisted of a number of algal, seagrass, and animal species. While nearly half (46%) of the collected material was so degraded as to be unidentifiable, 26% could be identified as red algae, 13% as seagrass, 12% as brown algae, 2% as green algae, and 1% as animals (see Table 2 for details). For the material that was identifiable, the following species were found. Red algae: Mostly *Furcellaria lumbricalis*, but also *Chondrus crispus*, *Polysiphonia* sp., and *Ceramium *sp. Brown algae: Almost only various *Fucus* spp. with small portions of *Ectocarpus* sp. and *Chorda filum*. Seagrass: Mostly *Zostera marina*, but also *Ruppia maritima*. Green algae: mostly *Cladophora* sp. and *Ulva intestinalis*. Animal remains: Mostly shells of *Mytilus edulis* and other bivalves.

### Wrack distribution and biomass

At site Tjälderviken in March 2021, the estimation of how much the wrack covered the beach was done using the beach length, approximately 200 m (Fig. 2a and b) and the wrack thickness, estimated by random depth measurements to be on average 49 cm thick (± 11SE, *n* = 12), with a minimum of 10 cm and a maximum of 110 cm (see Table 2 for details on thickness). The wrack covered the full length of the beach, with an average width of 13.3 m amounting to an area of 2667 m^2^ and a total volume of 1309 m^3^ beach wrack. This would be approximately 1026 metric tons of wrack as the Lova project reported an average density of 0.78 ton per 1 m^3^ of wrack in the area with an average mass of 0.38 tons m^−2^.

### Laboratory incubations and sampling

The laboratory incubations of beach wrack showed that isolated beach wrack had a high capacity to produce methane. The wrack gave emissions of between 0.2 and 0.7 mg CH_4_-C gDW^−1^ day^−1^, with an increase in emissions with temperature thereby showing the capacity of the wrack itself to emit CH_4_ (Fig. S1). At 15 °C, the emission was 0.24 (± 0.06SE), at 20 °C 0.49 (± 0.06 SE), and at 25 °C 0.66 (± 0.09 SE) mg CH_4_-C gDW^−1^ d^−1^. Recalculated as g CH_4_-C per ton fresh wrack and day this amounts to 25, 52, and 70 g CH_4_-C tonFW^−1^ d^−1^, respectively. This is within the same range as other laboratory emission estimations on beach wrack (e.g., Mission et al. 2020; Hansson 1983), and also considerably higher than the emissions from wrack on the beaches. As a comparison the highest field value recorded in this study (176 mg CH_4_-C m^−2^ d^−1^), recalculated per ton fresh biomass, would be 0.46 g CH_4_-C tonFW^−1^ d^−1^, using the estimated average of 0.38 tons fresh wrack m^−2^.

### Estimated yearly variation in methane emissions at site Tjälderviken

**Fig. 5:**
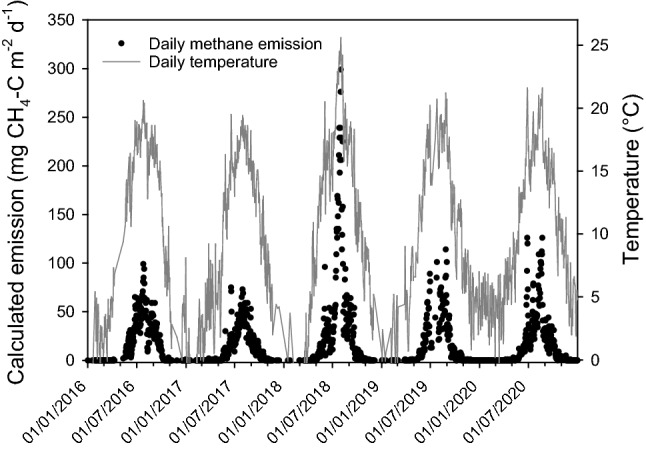
Estimated daily emissions of methane (CH_4_) from the Tjälderviken site calculated from meteorological temperature data (SMHI) and calculated temperature-dependent methane emission based on Fig. 4, showing the estimated daily emissions for the period 2016–2020

**Fig. 6:**
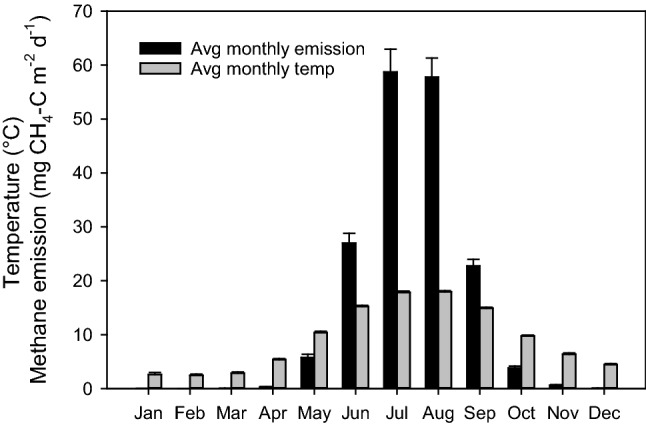
Monthly emissions of methane (CH_4_) as an average over five years, from the Tjälderviken site calculated from meteorological temperature data (SMHI) using the temperature dependence based on Fig. 4, showing the monthly means over the period 2016–2020

Using the average of all emission measurements at site Tjälderviken (at all temperatures and water contents), the average diel temperature data, and the correlation between emissions and temperatures (as per Fig. 4), we calculated expected emissions at different temperatures over 5 years (2016–2020) (Fig. 5) assuming that the general water content of the wrack had stayed similar over that period. As expected, the calculated emissions were negligible during the colder months, but increased sharply around May and then nearly disappeared around October (Fig. 6). During summers with higher than normal temperatures (e.g., during 2018), the methane emission levels rose drastically. The average yearly emissions calculated over the 5-year period 2016–2020 was 17.9 ± 3.16 mg CH_4_-C m^−2^ d^−1^, while the average monthly summer emissions for the same period reached 59 mg CH_4_-C m^−2^ d^−1^, with yearly peaks at the warmest days of 100–300 mg CH_4_-C m^−2^ d^−1^. The number of days in each year with an average diel temperature above 20 °C were 0 in 2015, 2 in 2016, 0 in 2017, 29 in 2018, 5 in 2019, and 13 in 2020. The highest average was recorded in 2018 with an average diel temperature of 25.6 °C on August 2.

## Discussion

This study demonstrates that decomposing macrophyte beach wrack emits large amounts of methane (up to 176 mg CH_4_-C m^−2^ day^−1^) to the atmosphere when it accumulates on beaches under wet conditions. Overall, there were large variations in emissions between different collection times and sites. However, emissions clearly correlated positively with temperature and with the placement of the wrack material: the wrack closest to the sea that were waterlogged emitted drastically higher levels than the material higher up on the beach that had been drained of water.

The highest atmospheric emissions of methane were measured where wrack was dense and waterlogged and during warm summer months. This agrees with previous works from similar habitats, reporting the most important factors controlling methane fluxes in coastal systems to be organic matter content and quality as well as oxygen availability (Al-Haj and Fulweiler 2020) and temperature (e.g., Zeikus and Winfrey 1976; Dunfield et al. 1993; Roth et al. 2022). In waterlogged soils or wrack at the waterfront, microbial degradation of organic matter quickly uses up ambient free oxygen (Wegner 2010), and the slow diffusion of gases in water will drastically limit the transport of oxygen from the atmosphere (Beer et al. 2014), thereby creating anaerobic conditions that favor methanogenesis (Barnes and Goldberg 1976). Temperature influences microbially mediated biogeochemical processes including methanogenesis (Zeikus and Winfrey, 1976; Westermann et al. 1989; Dunfield et al. 1993; Van Bodegom and Stams 1999; Sanz-Lázaro et al. 2011), and changes in temperature can result in rapid changes in methane production (Segers, 1998; Chin et al. 1999; Van Bodegom and Stams, 1999; Høj et al. 2008; Borges et al., 2018). This is explained partly by a direct effect on the process, where methane production has a Q10 (i.e., a relative increase in activity after an increase in temperature of 10 °C) of 1.3–28 (Dunfield et al. 1993; Segers 1998; Van Hulzen et al. 1999) and partly by a temperature-driven shift in composition and activity of the microbial community (Pender et al. 2004; Høj et al. 2008; Conrad et al. 2009). Thus, additional studies on the microbial community structure within the wrack could help to further explain the large variations in methane emissions among the tested sampling sites.

Not all thick and wet beach wrack emitted high levels of methane. The material at Snäckviken, sampled when the temperature was high (21 °C), did not emit any measurable levels of methane even though the beach wrack was deposited in a thick (15–30 cm) layer. The reason was most likely that the wrack was fresh, porous, and to a large degree not yet decomposing. Fresh beach wrack can metabolize and produce oxygen for long time periods, even as fractionated, and beach kelp has been shown to be photosynthetically active for up to 56 days (Frontier et al. 2021). Although we did not perform any oxygen profiling of this material, it was assumed that it was well oxygenated and, thus, not methanogenic (cf. Lyu et al. 2018).

To estimate the potential climate mitigation benefit of removing beach wrack from accumulation beaches, we calculated the yearly variations in methane emission for the years 2016–2020. The average yearly emission over this period was 17.9 ± 3.2 mg CH_4_-C m^−2^ day^−1^ or 6.5 ± 1.1 g CH_4_-C m^−2^ year^−1^ and varied greatly, both between months and years. The calculated monthly averages from the Tjälderviken site, displayed in Fig. 6, show that methane emissions are increasing drastically in the beginning of the summer. The emissions then peaks in the warmest summer months, July and August, with average emissions reaching 59 mg CH_4_-C m^−2^ day^−1^. This is in the same range as emissions from northern lakes and wetlands, the largest natural source of atmospheric methane (Turetsky et al. 2014; Jansen et al. 2019), and considerably higher than those reported from beach wrack seagrass at the High Adriatic coast where emission during summer was estimated to 0.14 mmol CH_4_ m^−2^ day^−1^ or 1.7 mg CH_4_-C m^−2^ day^−1^ (Mission et al. 2021), but in the lower part of the range of methane emission from rice paddy fields (Minami 1994; Minami and Neue 1994). Using the calculated average yearly emissions of 17.9 mg CH_4_-C m^−2^ day^−1^, we estimate that Tjälderviken’s wrack alone releases 23 kg CH_4_-C to the atmosphere every year. Calculated as eCO_2_, the methane emissions from wrack in the bay releases 3.9 kg eCO_2_ per year per meter of beach and in total the methane from this bay amounts to a release of 790 kg eCO_2_ to the atmosphere every year. These emissions could be nearly eliminated if the wrack was collected and piled into heaps and then used as fertilizer.

Our calculation shows greatly increased emissions during the recorded heatwaves in the summers of 2018 and 2020. In the summer of 2018, the prolonged heatwave resulted in 29 days with average diel temperatures above 20 °C, occasionally reaching average diel temperatures over 25 °C. As a result, the calculated methane emissions from Tjälderviken peaked at about three times higher levels than during the same periods of other years. Global warming might further increase methane emissions from beach wrack in the area of the present study. Gotland County is estimated to increase its yearly average temperature by 3 degrees according to IPCC Representative Concentration Pathways (RCP) 4.5 and by 5 degrees according to RCP 8.5 until the end of the century (Persson et al. 2015). The greatest warming is expected during the summer, when the number of hot days will increase dramatically, and RCP 8.5 shows an annual average value of over 30 consecutive days with daily average temperatures of over 20 °C by the end of the century. The weather will also be wetter as the annual average precipitation is expected to increase with 20–30% toward the end of the century with more frequent heavy rainfalls (Persson et al. 2015). Thus, a larger portion of beach wrack might be moist to a degree where it starts to emit significant amounts of methane. Also changes in wave actions due to changing weather patterns will affect both the moisture and accumulation of beach wrack (Suursaar et al. 2014). As the influence of changing weather patterns on the moisture of the beach wrack thus are difficult to predict, we did not include them in the calculations, but as the emissions from beach wrack were positively correlated to both increases in temperature and moisture, it is highly likely that methane emissions will increase considerably in future, especially in marine areas of the world characterized by low salinity, such as the Baltic Sea.

Beach wrack deposits on beaches also leak nutrients back to the sea when decomposing. The release will depend on the time needed for decomposition, which ranges from less than a day to about a month (Mews et al. 2006) and the nutrient content of the wrack. It has been shown that nutrient levels of shore water can be strongly correlated with beach wrack biomass, suggesting a direct export of nutrients from decomposing wrack (Dugan et al. 2011). Although nutrient leaking was not studied in the present study, beach wrack from the region of the present study has been reported to contain an average of 19 kg N and 2 kg P for every ton of wrack (Sinha et al. 2022), so if the Tjälderviken site alone contains ~ 500 tons of beach wrack, it would mean that cleaning the beach from this amount of wrack could prevent around 9500 kg N and 1000 kg P to reach the coastal water. This would be a considerable advantage as the Baltic waters are already eutrophic (Murray et al. 2019). Also, the deposition of collected beach wrack on farmlands, as is a requirement in Gotland County in order to get funding from LoVa, will also reduce the need for fertilizers on the farmland. This will also lead to an increased organic matter content in farmlands and thus a prolonged CO_2_ storage in the soils which is enhancing the climate change mitigation effect of beach wrack collection (Chubarenko et al. 2021). Considering the high levels of methane emissions, it is important to conduct further studies on the dynamics of the wrack and the environmental drivers of methane production in other environmental settings and during all seasons. To deal with the large amounts of wrack that has to be removed it would be useful to apply a life cycle assessment modeling for assessing alternative seagrass management solutions (Mainardis et al. 2021).

We conclude that there are large benefits for the environment to remove waterlogged wrack from beaches. Without sufficient wrack gathering, more beaches would become like Tjälderviken with constant accumulations of decaying wrack causing high summer methane emissions to the atmosphere, which most probably will increase with global warming.

## Supplementary Information

Below is the link to the electronic supplementary material.

Supplementary file1 (PDF 692 KB)
