# Supplementary material for: Methane emissions from macrophyte beach wrack on Baltic seashores
Source: Ambio. 2022 Aug 27;52(1):171–81. doi: 10.1007/s13280-022-01774-4 (PMC9666566; doi:10.1007/s13280-022-01774-4)
Supplement: Supplementary file 1 — Supplementary file1 (PDF 692 KB) [file 13280_2022_1774_MOESM1_ESM.pdf]

Ambio

Supplementary Information

This supplementary information has not been peer reviewed.

Title: Methane emissions from macrophyte beach wrack on Baltic seashores

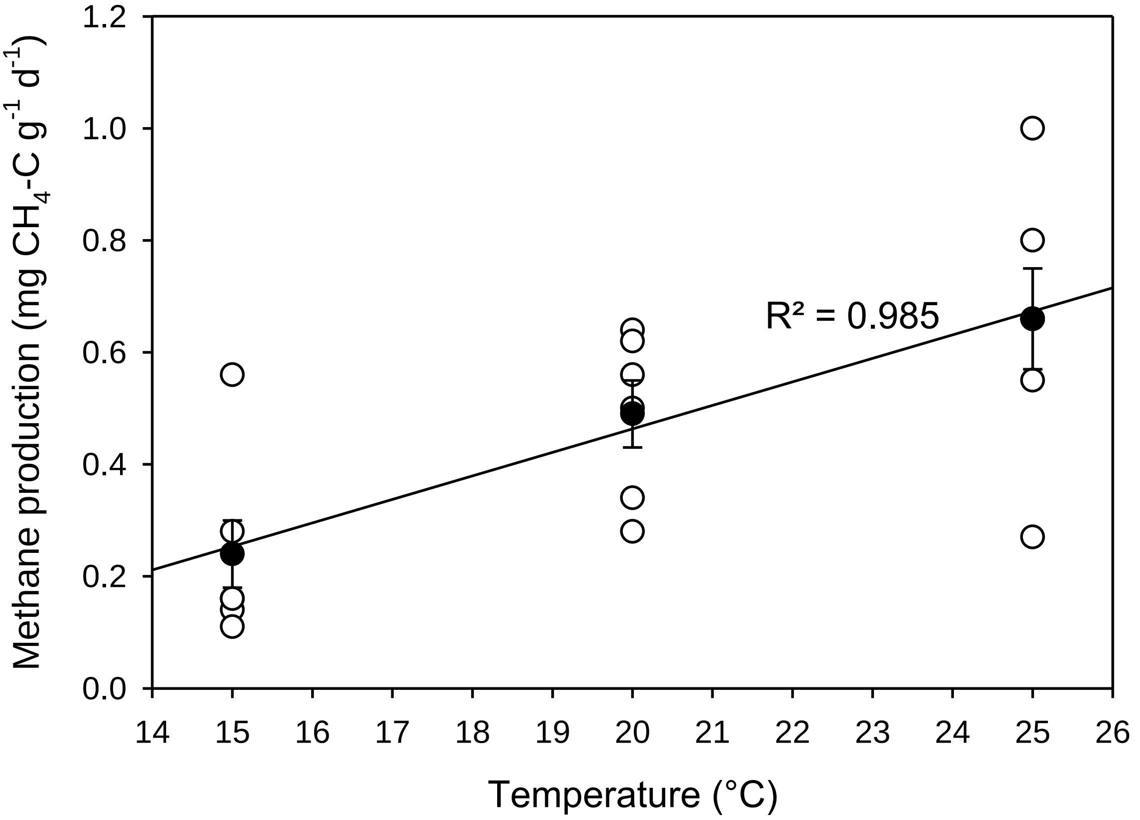

Figure S1. *Methane production in the laboratory from beach wrack sampled at site "Tjälderviken" on September 15 and incubated at different temperatures. Black circles are averages at each temperature (±SE, n=6), open circles are individual values.*
